# Long-term follow-up results of patients with left bundle branch pacing and exploration for potential factors affecting cardiac function

**DOI:** 10.3389/fphys.2022.996640

**Published:** 2022-09-15

**Authors:** Qingyun Hu, Wenzhao Lu, Keping Chen, Yan Dai, Jinxuan Lin, Nan Xu, Jingru Lin, Ruohan Chen, Yao Li, Chendi Cheng, Yu’an Zhou, Shu Zhang

**Affiliations:** ^1^ Department of Cardiology, Fuwai Hospital, National Center for Cardiovascular Diseases, Chinese Academy of Medical Sciences and Peking Union Medical College, Beijing, China; ^2^ Department of Echocardiography, Fuwai Hospital, National Center for Cardiovascular Diseases, Chinese Academy of Medical Sciences and Peking Union Medical College, Beijing, China

**Keywords:** left bundle branch pacing, long-term follow-up, pacing performance, echocardiographic evaluation, cardiac function

## Abstract

**Background:** Left bundle branch pacing (LBBP) is an alternative strategy for His bundle pacing (HBP). This study aimed to analyze the long-term performance of LBBP and the potential factors affecting long-term cardiac function.

**Methods:** Patients with LBBP were continuously enrolled from January 2018 to August 2020. Pacing parameters, electrocardiogram (ECG), and echocardiography were collected. The anatomic position of LBBP leads was described by echocardiographic and fluoroscopic parameters.

**Results:** A total of 91 patients with a median follow-up of 18 months were enrolled. Most patients maintained stable pacing parameters during follow-up. The intra-septal position of the 3830 lead also remained stable as the distance from the lead tip to the left surface of the ventricular septum was 0.4 (0, 1.4) mm. The overall level of left ventricular ejection fraction (LVEF) slightly increased. 59 patients had improved LVEF (∆LVEF > 0), while 28 patients had unchanged or reduced LVEF (∆LVEF ≤ 0). The declines of baseline LVEF, ∆ Paced QRSd, and corrected longitudinal distance (longit-dist) of lead-implanted site correlated with LVEF improvement, and these three factors had negative linear correlations with ∆LVEF. Patients with tricuspid valve regurgitation (TVR) deterioration had longer follow-up duration (20.5 vs. 15.0 months, *p* = 0.01) and shorter Lead-TVA-dist (18.6 vs. 21.6 mm, *p* = 0.04) than those without TVR deterioration.

**Conclusion:** Patients with LBBP generally remained stable in pacing performance, anatomic lead positions, and cardiac function in long-term follow-up. Baseline LVEF, ∆ Paced QRSd, and corrected longit-dist might be associated with potential LVEF decrease, which required further confirmation.

## 1 Introduction

His bundle pacing (HBP) is considered the most physiological form of pacing, as it captures the intrinsic conduction system and delivers physiological ventricular activation (Lustgarten et al., 2015; Abdelrahman et al., 2018; Arnold et al., 2018; Sharma et al., 2018; Vijayaraman et al., 2018). However, HBP still has limitations, such as a steep learning curve, elevations of pacing threshold, a low R wave amplitude, and complicated pacemaker programming (Keene et al., 2019). Left bundle branch pacing (LBBP) is an alternative near-physiological pacing method that is considered to conquer the above shortcomings of HBP (Zhang et al., 2019). It has been shown to achieve favorable left ventricular (LV) electrical and mechanical synchrony similar to HBP (Hou et al., 2019). Although short-term and relatively long-term safety and feasibility have been demonstrated in several studies (Chen et al., 2019; Vijayaraman et al., 2019; Padala et al., 2020; Sharma et al., 2021; Su et al., 2021), these studies lacked detailed descriptions of the anatomical position of the LBBP lead in the ventricular septum and potential factors affecting patients’ cardiac function after LBBP.

This study aimed to provide the long-term follow-up data of patients who received LBBP in Fuwai Hospital and explore the factors associated with potential changes in cardiac function while describing the anatomical position stability of LBBP lead.

## 2 Materials and methods

### 2.1 Study population and follow-up

This study is a prospective study. Patients who indicated permanent pacing according to current AHA/ACC/HRS guidelines and underwent successful LBBP implantation from January 2018 to August 2020 were prospectively followed up. Finally, those who had a pre-operative left ventricular ejection fraction (LVEF) ≥ 40% and a follow-up time ≥ 9 months with integral echocardiographic evaluation were included for analysis.

Patients with the following conditions were excluded: 1) receiving triple-chamber pacemaker implantation; 2) upgrading to conventional or LBBP-optimized cardiac resynchronization therapy (CRT) within 9 months.

Successful LBBP was defined as follow: the paced QRS morphology manifests as a right bundle branch block (RBBB) pattern; recording an LBB potential; transition from non-selective LBBP (ns-LBBP) to selective LBBP (s-LBBP) during threshold testing; or transition from left ventricular septal pacing (LVSP) to ns-LBBP or the stimulus to R wave peak time in V6 ECG lead (V6RWPT) was abruptly shortened (≥10 ms) at a higher output (5 V/0.4 ms) and (or) remained short (≤80 ms) and constant at different outputs (Li et al., 2019).

Patients were recommended for outpatient follow-up at 1, 3, and subsequently every 6 months. If there were any problems or discomforts about pacing or arrhythmia, additional clinic visits would be required.

The study was approved by the Ethics Committee of Fuwai Hospital (Approval No. 2019-1149) and obeyed the Declaration of Helsinki. Patients had signed written informed consents for pacemaker implantation and clinical data use before the operations.

### 2.2 Implantation procedure of left bundle branch pacing

We used the trans-ventricular-septal approach to achieve LBBP as previously described (Chen and Li, 2019). Briefly, the 3830 pacing lead (SelectSecure™, Model 3830, Medtronic, Minneapolis, MN, United States) was located on the right side of the interventricular septum (IVS) *via* the C315HIS sheath (Medtronic, Minneapolis, MN, United States) in the right anterior oblique (RAO) 30° fluoroscopic view; unipolar (tip) pacing with 2.0 V/0.5 ms was applied to select a targeted site and confirm the excellent contact between the lead and the septum. Then the lead was screwed perpendicularly into the IVS and towards the left side of IVS (left bundle branch area, LBB area). Advancing the lead while monitoring the paced QRS morphology until the criteria for successful LBBP (for details, see Section 2.1) were achieved.

During the procedure, 12-lead ECG and intracardiac electrogram (EGM) were displayed and recorded in real-time by the Bard system (Bard LabSystem Pro EP Recording System 2.4a.65.0, MA, United States). The procedure was terminated if the paced QRS morphology failed to meet the criteria for successful LBBP (for details, see Section 2.1) within five attempts.

### 2.3 Pacing and electrocardiogram parameters

**FIGURE 1:**
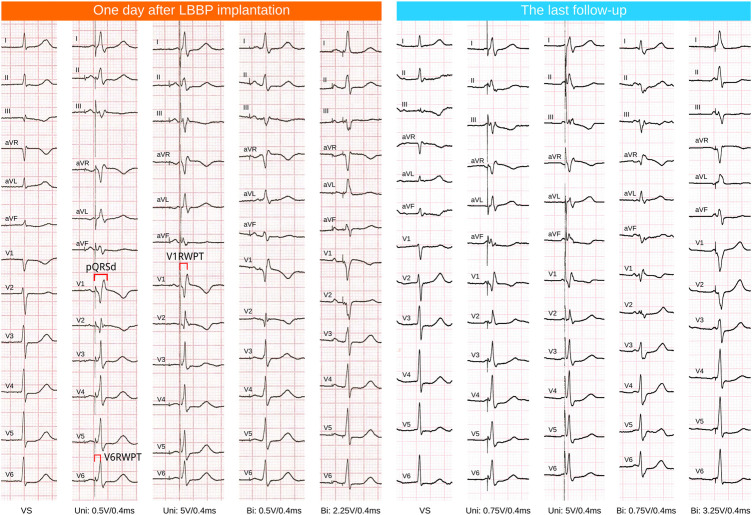
ECG follow-up of one case. LBBP, left bundle branch pacing; VS, ventricular sensing or intrinsic rhythm; Uni, unipolar tip pacing; Bi, bipolar pacing, anodal ring capture was observed at relatively high outputs (the QRS morphology showed the absence of a R’ wave in ECG lead V1).

Pacemaker programming and ECG inspection were performed on the day after operations and the clinic follow-up (Figure 1). The last follow-up date was included in the analysis. Pacing parameters included R wave amplitude, pacing threshold, and impedance. ECG data included intrinsic QRS duration (QRSd), paced QRSd, V6RWPT, the stimulus to R wave peak time in V1 ECG lead (V1RWPT), and V6-V1 interpeak interval (V1RWPT-V6RWPT). All the above ECG parameters were measured during unipolar pacing at the LBB capture threshold in VVI mode at 10 bpm above the intrinsic rate (VVI at 60 bpm was used for those without intrinsic ventricular rhythm or complete atrioventricular block). At least three paced QRS complexes were measured, and the average was taken (Lin et al., 2021).

### 2.4 Echocardiographic parameters

**FIGURE 2:**
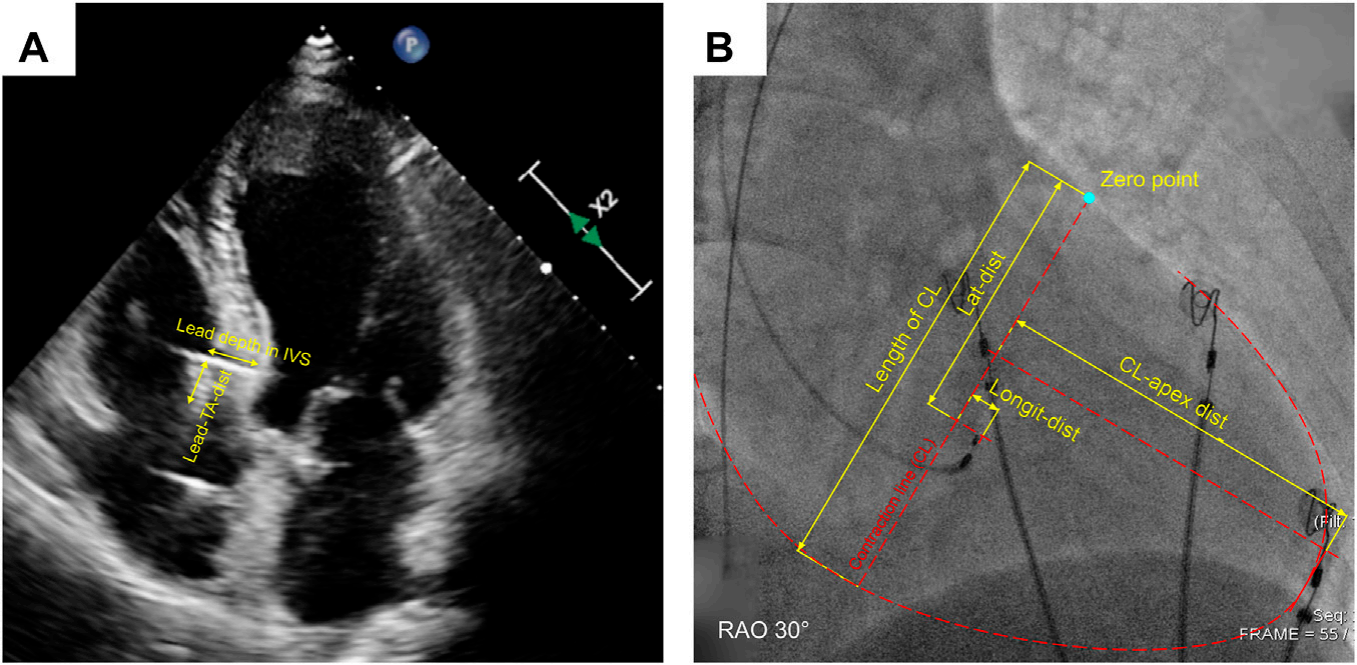
Measurement of echocardiographic **(A)** and fluoroscopic distance parameters **(B)**. IVS, interventricular septum; Lead-TA-dist, distance from the lead-implanted site on the right surface of IVS to the septal leaflet of tricuspid annulus; CL, contraction line; CL-apex-dist, distance from CL to apex; Longit-dist, longitudinal distance; Lat-dist, lateral distance.

Parameters of the anatomic position of 3830 lead for all enrolled patients were verified by echocardiography at the last follow-up, including lead depth in IVS (from the lead-implanted site on the right surface of IVS to the lead tip), IVS thickness at the lead-implanted site, distance from lead tip to the left surface of IVS (tip-to-LVS), and the distance from the lead-implanted site on the right surface of IVS to the septal leaflet of tricuspid annulus (Lead-TA-dist) (Figure 2A). These parameters were measured during the ventricular end-diastolic phase in the apical three/four-chamber and parasternal short-axis views. Other functional parameters were also measured at baseline and follow-up, including LVEF (evaluated with 2D biplane modified Simpson method), left ventricular end-diastolic dimension (LVEDD), and degrees of tricuspid valve regurgitation (TVR), TVR flow speed, and TVR pressure gradient. The degree of TVR was evaluated with the proximal isovelocity surface area (PISA) method and semi-quantitatively assessed in four classes (none, PISA radius ≤ 5 mm mild, 6∼9 mm moderate, >9 mm severe). TVR deterioration was defined as the TVR degree elevated by at least one class. The ultrasonic machine (EPIQ 7C, Philips Inc.) was used in all patients.

### 2.5 Fluoroscopic distance parameters of lead-implanted sites

We have invented a coordinate system to describe the distribution of the lead-implanted sites quantitatively (Lu et al., 2021). The definitions of contraction line (CL), distance from CL to apex (CL-apex-dist), longitudinal distance (longit-dist), lateral distance (lat-dist), corrected longit-dist, and corrected lat-dist were described in the Supplementary Materials along with the measuring and conversion methods (Supplementary Table S1) and illustrated in Figure 2B. Image measurement was performed with at least three-time repeats on the LibreCAD 2.1.3 software, and the average was taken.

### 2.6 Statistical analysis

Continuous variables were presented as mean ± SD (normal distribution) or median (IQR) (non-normal distribution), while categorical variables were presented as numbers and percentages. Data between baseline and follow-up were compared using the paired-sample *t*-test (normal distribution) or the Wilcoxon signed-rank test; Inter-group comparisons were made by the independent-sample *t*-test or the Wilcoxon rank-sum test. Categorical variables were compared by the Chi-square test or Fisher’s exact test. Linear correlations between variables were assessed by linear regression. Changes in variables from baseline to the last follow-up were presented as “∆variable”.

After univariate comparison, variables with *p* < 0.15 were considered as potential confounding factors and further screened by the logistic least absolute shrinkage and selection operator (Lasso) regression model, which is a shrinkage method to select the more relevant and explainable predictors from numerous variables with potential multicollinearity while avoiding overfitting (Falconer, 2011). The higher the lambda (λ) value was, the more strict the penalty was, while fewer variables remained. The largest *λ* value within one standard error (1SE) of the minimum binomial deviance was used for variable selection to generate the more simplified but still representative model. The SEs of variable coefficients in the Lasso model were estimated by the bootstrapping re-sampling algorithm (500 replicates). All tests were two-sided. *p* < 0.05 was considered statistically significant.

## 3 Results

### 3.1 Study population

A total of 176 patients underwent successful LBBP within the time window of patient enrollment, among whom 26 had the baseline LVEF < 40% (24 received CRT device and 2 upgraded to CRT) and 59 had a follow-up time of fewer than 9 months (Supplementary Figure S1). Eventually, 91 patients were included for analysis.

The median follow-up time was 18 (13, 23) months. The baseline characteristics are summarized in Table 1. Distance parameters of the 3830 lead under echocardiography and fluoroscopy are summarized in Table 2. Echocardiography revealed that the lead depth in IVS was 10.8 ± 2.1 mm, the IVS thickness at lead-implanted sites was 11.7 ± 2.2 mm, and the median tip-to-LVS was 0.4 (0, 1.4) mm. Despite the resolution limitation of the ultrasound imaging, it was reasonable to consider that the tip of the leads kept stable at the sub-endocardial area of LVS during the follow-up period.

**TABLE 1:** Baseline clinical and demographic characteristics.

Characteristics	Enrolled patients (*n* = 91)
Age (years)	67 (58.5, 73.0)
Male sex	39 (42.9)
Pacing indications	
Sick sinus syndrome	36 (39.6)
Atrioventricular block	50 (54.9)
AF with bradycardia	5 (5.5)
Comorbidity	
Coronary heart disease	26 (28.6)
Hypertension	51 (56.0)
Diabetes mellitus	19 (20.9)
Hyperlipidemia	38 (41.8)
Stroke history	12 (13.2)
Intrinsic QRS duration (ms)	89.4 (84.4, 96.9)
Intrinsic QRS duration > 120 ms	13 (14.3)
Intrinsic QRS morphology	
Narrow	77 (84.6)
Right bundle branch block	10 (11.0)
Left bundle branch block	4 (4.4)

Data was presented as n (%) or median (IQR).

**TABLE 2:** Distance parameters of the 3830 lead under echocardiography and fluoroscopy.

Distance parameters	All patients (*n* = 91)
Echocardiography	
Lead depth in IVS (mm)	10.8 ± 2.1
IVS thickness (mm)	11.7 ± 2.2
Lead tip to LVS (mm)	0.4 (0, 1.4)
Lead-TA-dist (mm)	20.8 ± 6.7
Fluoroscopy	
Length of CL (mm)	147.5 (140.1, 155.7)
CL-apex-dist (mm)	118.5 ± 12.7
Longit-dist (mm)	25.6 ± 11.6
Lat-dist (mm)	79.2 ± 13.4
Corrected longit-dist (mm)	25.6 ± 11.1
Corrected lat-dist (mm)	79.4 ± 13.3

Data was presented as mean ± SD or median (IQR). IVS, interventricular septum; LVS, left surface of ventricular septum; Lead-TA-dist, distance from the lead-implanted site on the right surface of interventricular septum to the septal leaflet of tricuspid annulus; CL, contraction line; CL-apex-dist, distance from CL to apex; Longit-dist, longitudinal distance; Lat-dist, lateral distance.

### 3.2 Comparisons of baseline and follow-up characteristics

Comparisons between baseline and follow-up characteristics are given in Table 3. During follow-up, the threshold and the R wave amplitude mildly increased but the pacing impedance decreased more prominently [750 (643, 880) vs. 399 (361, 427) ohm, *p* < 0.001]. However, the changes of pacing parameters were still within the clinically acceptable range, and the pacing performance could be considered stable. Regarding the ECG parameters, although V6RWPT (68.1 ± 9.7 to 71.1 ± 9.9 ms, *p* < 0.001) and V1RWPT (100.9 ± 11.1 to 103.6 ± 10.7 ms, *p* = 0.004) during follow-up were significantly prolonged compared with baseline, the magnitude of these changes were negligible; the paced QRSd and V6-V1 interpeak interval remained stable throughout follow-up. Echocardiographic parameters also remained stable during follow-up. Despite the increment was small, LVEF did increase significantly [63.0 (60, 65) % to 65.0 (61.0, 68.5) %, ∆LVEF = 2.5% ± 6.2%].

**TABLE 3:** Comparison of pacing/ECG and echocardiographic parameters between baseline and follow-up.

Variables	Baseline (*n* = 91)	Follow-up (*n* = 91)	*p* value
Pacing/ECG parameters			
R wave amplitude (mV)	12.0 (7.8, 16.4)	15.7 (12.0, 20.0)	<0.001
Pacing impedance (ohm)	750 (643, 880)	399 (361, 427)	<0.001
Threshold (V/0.4 ms)	0.6 ± 0.4	1.03 ± 0.6	<0.001
Paced QRS duration (ms)	104.7 ± 11.9	105.7 ± 12.5	0.29
V6RWPT (ms)	68.1 ± 9.7	71.1 ± 9.9	<0.001
V1RWPT (ms)	100.9 ± 11.1	103.6 ± 10.7	0.004
V6-V1 interpeak interval (ms)	32.8 ± 10.0	32.6 ± 10.7	0.59
Echocardiography			
LVEF (%)	63.0 (60, 65)	65.0 (61.0, 68.5)	<0.001
LVEDD (mm)	47.0 (45, 50)	46.4 (44, 50)	0.06
TVR severity grades			
None	31 (34.0)	23 (25.3)	0.26
Mild	29 (31.9)	36 (39.6)	0.35
Moderate	23 (25.3)	16 (17.6)	0.28
Severe	8 (8.8)	16 (17.6)	0.13
TVR flow speed (m/s)	2.3 (0, 2.6)	2.2 (0, 2.5)	0.72
TVR pressure gradient (mmHg)	21.2 (0, 27)	20.0 (0, 24.5)	0.58

Data was presented as n (%), mean ± SD, or median (IQR). ECG, electrocardiogram; V6RWPT, stimulus to R wave peak time in V6 ECG lead; V1RWPT, stimulus to R wave peak time in V1 ECG lead; LVEF, left ventricular ejection fraction; LVEDD, left ventricular end-diastolic dimension; TVR, tricuspid valvular regurgitation.

Compared with baseline, at the last follow-up, seven patients’ V6RWPT (7.8%) and nine patients’ paced QRSd (9.9%) were prolonged more than 10 ms at the LBB capture thresholds, while four patients (4.4%) lost the typical RBBB pattern in V1 ECG lead.

### 3.3 Comparison between patients with improved and unchanged/reduced left ventricular ejection fraction

During the follow-up, 59 (67.8%) patients had improved LVEF (∆LVEF > 0), while 28 (32.2%) patients had unchanged or reduced LVEF (∆LVEF ≤ 0). Using ∆LVEF = ±5% as the cut-offs, the number of patients was 29 (33%), 48 (55.2%), 10 (11.5%) in the ranges of ∆LVEF ≥ 5%, ∆LVEF from −5% to 5%, and ∆LVEF < −5%, respectively. The result indicated that the cardiac function of most patients with successful LBBP remained stable, while a small proportion had a significant reduction in LVEF (∆LVEF < −5%).

Differences between patients with ∆LVEF > 0 and ∆LVEF ≤ 0 were shown in Supplementary Table S2. Baseline LVEF [62 (60, 65) % vs. 65 (62, 65.3) %, *p* = 0.003], ∆Paced QRSd [−0.4 (−5.1, 4.7) vs. 2.5 (−0.7, 9.3) ms, *p* = 0.006], ∆V6RWPT [2.0 (−0.6, 5.6) vs. 5.1 (0.4, 6.9) ms, *p* = 0.04], ∆V1RWPT (1.2 ± 7.3 vs. 4.6 ± 6.0 ms, *p* = 0.03) were significantly lower and the corrected longit-dist (23.4 ± 11.0 vs. 29.1 ± 10.5 mm, *p* = 0.02) was shorter in patients with ∆LVEF > 0. The results indicated that patients with improved LVEF might have worse baseline cardiac function (although the difference is small), higher stability of ECG depolarization parameters, and closer lead-implanted sites towards the CL. These were the potential correlated factors for LVEF changes in patients with long-term LBBP.

### 3.4 Correlative factors for the change of left ventricular ejection fraction

**FIGURE 3:**
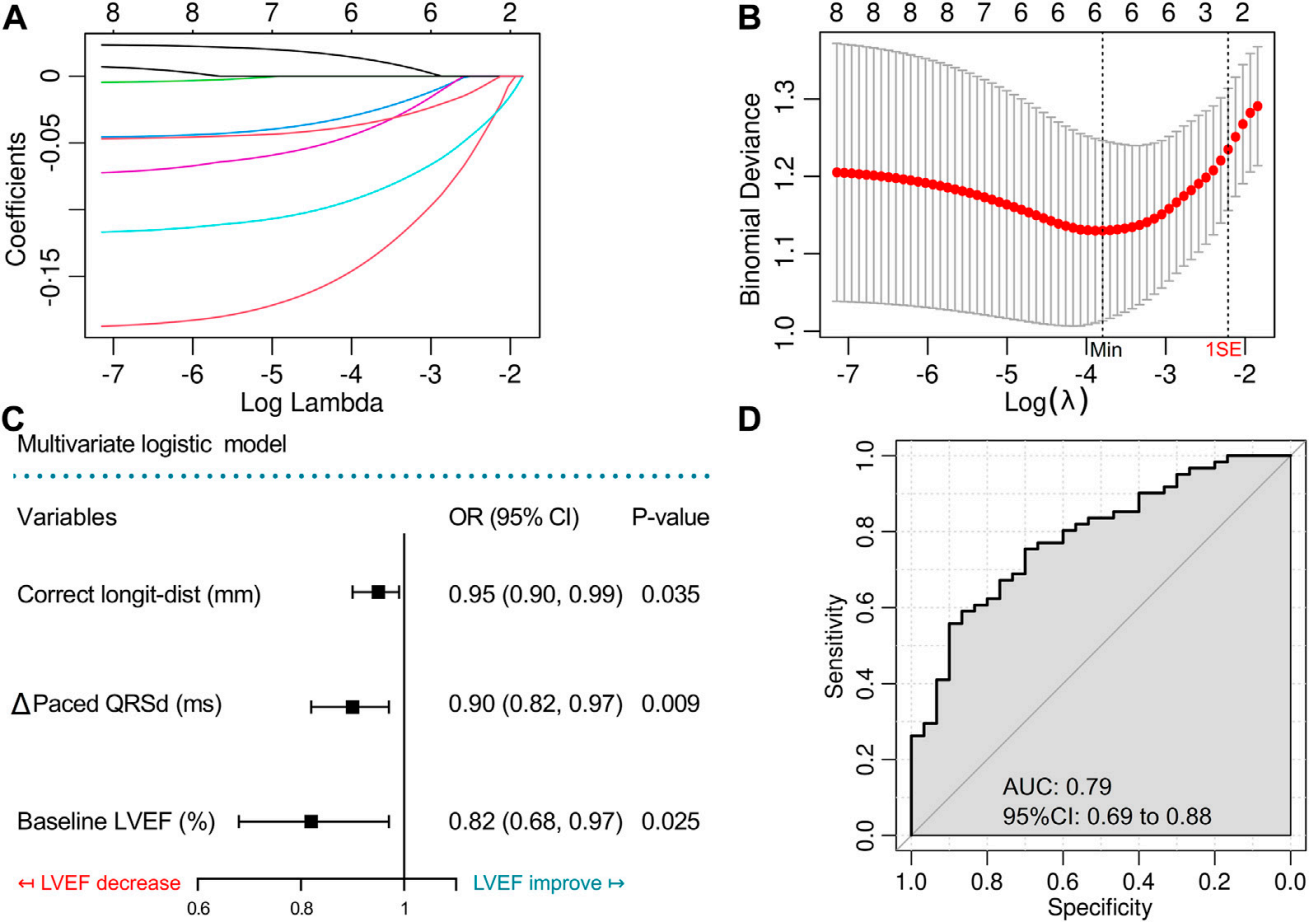
Variable screening by Lasso regression model and construction of the multivariate logistic model with ROC evaluation. **(A)** Lasso regularization of the binomial logistic model, the higher the lambda value was, the heavier was the penalty, while the remaining variables were less. **(B)** Cross-validation of the Lasso model to determine the optimal lambda value. **(C)** Forest plot shows the results of the final multivariate logistic model. **(D)** Receiver operating characteristic (ROC) curve to evaluate the final multivariate logistic model. Longit-dist, longitudinal distance; QRSd, QRS duration; LVEF, left ventricular ejection fraction; AUC, area under curve.

To investigate the independent ∆LVEF related factors, we recruited variables with *p* < 0.15 in the univariate comparison between ∆LVEF > 0 and ∆LVEF ≤ 0 groups (including age, baseline paced QRSd, baseline LVEF, ∆Paced QRSd, ∆V6RWPT, ∆V1RWPT, lead-TA-dist, corrected longit-dist) in multivariate analysis. Variables were screened by the Logistic-Lasso regression model. As shown in Figure 3A, with the increase of λ value, coefficients of more and more variables shrunk to zero, and the remaining variables became fewer. When the binomial deviance was minimized [*λ* = 0.02, log (λ) = −3.91], the model still contained six variables without enough simplification. Finally, the largest *λ* value [*λ* = 0.097, log (λ) = −2.33] within 1SE of the minimum binomial deviance (Figure 3B) was applied to generate the less complicated model containing three variables, including baseline LVEF, ∆ Paced QRSd and corrected longit-dist (Table 4).

**TABLE 4:** Screen variables by the Lasso regression model.

Variables	Coefficients (bootstrap SE)
	λ = 0.097, log (λ) = −2.33
Age (years)	0
Baseline LVEF (%)	−0.031 (0.062)
Baseline paced QRSd (ms)	0
∆Paced QRSd (ms)	−0.029 (0.031)
∆V6RWPT (ms)	0
∆V1RWPT (ms)	0
Lead-TA-dist (mm)	0
Corrected longit-dist (mm)	−0.003 (0.017)

The Lasso regression model enrolled variables with the *p* values < 0.15 in the comparison between patients with improved and decreased LVEF. The optimal lambda value of 0.097 was chosen which was one-fold standard error (1 SE) away from the lambda of the minimum binomial deviance (*λ* = 0.020). Variables with beta equaling to 0 was excluded. LVEF, left ventricular ejection fraction; QRSd, QRS duration; ∆Paced QRSd/V6RWPT/V1RWPT, changes of QRSd/V6RWPT/V1RWPT from baseline to follow-up; V6RWPT, stimulus to R wave peak time in V6 lead; V1RWPT, stimulus to R wave peak time in V1 lead; Lead-TA-dist, distance from the lead-implanted site on the right surface of interventricular septum to the septal leaflet of tricuspid annulus; Longit-dist, longitudinal distance.

**FIGURE 4:**
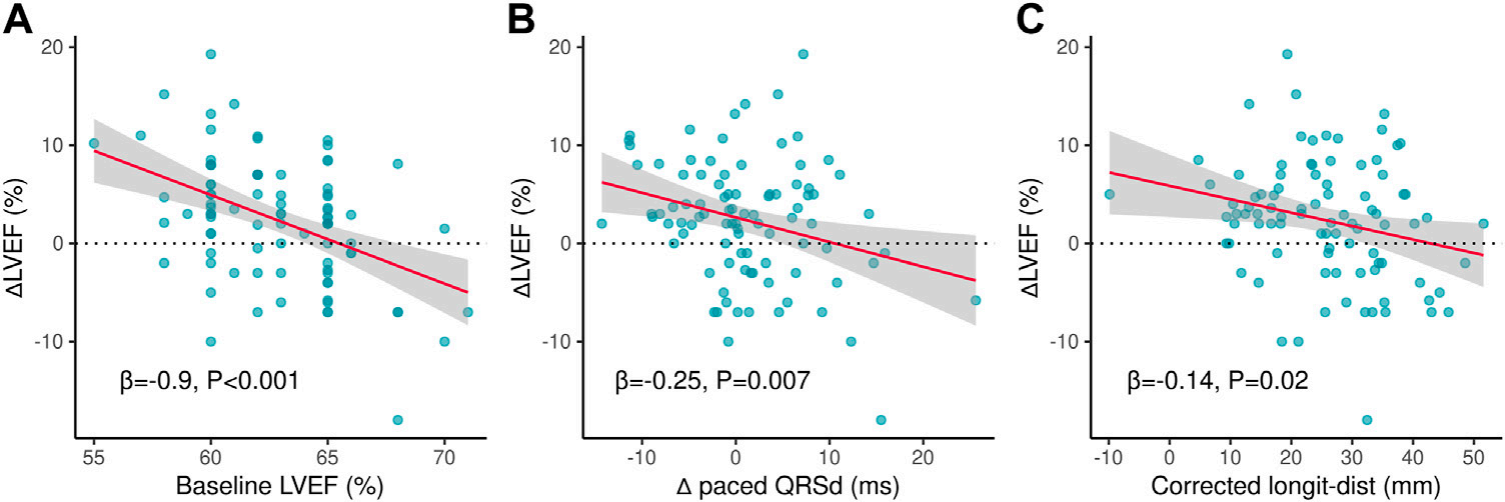
Linear correlated variables of ∆LVEF. **(A)** Negative linear correlation between ∆LVEF and baseline LVEF. **(B)** Negative linear correlation between ∆LVEF and ∆paced QRSd. **(C)** Negative linear correlation between ∆LVEF and corrected longit-dist. ∆LVEF, changes of left ventricular ejection fraction from baseline to follow-up; ∆paced QRSd, changes of paced QRS duration from baseline to follow-up; Longit-dist, longitudinal distance.

The three variables were then incorporated into a simplified logistic regression model, revealing that the declines in these variables correlated to LVEF improvement (Figure 3C). The receiver operating characteristic (ROC) curve (AUC = 0.79, 95%CI 0.69–0.88) indicated the favorable efficacy of this triple-variate model (Figure 3D). These three variables’ data were conformed to normal distribution (Supplementary Figure S2), and negative linear correlations were demonstrated between the three variables and ∆LVEF (Figures 4A–C).

### 3.5 Correlative factors for tricuspid valve regurgitation deterioration

In the present study, the comparison between patients with and without TVR deterioration was described in Supplementary Table S3. Patients with TVR deterioration had significantly longer follow-up duration [20.5 (17.0, 24.0) vs. 15.0 (12.5, 21.5) month, *p* = 0.01] and shorter Lead-TVA-dist [18.6 (14.0, 23.0) vs. 21.6 (18.9, 25.8) mm, *p* = 0.04] than those without TVR deterioration (Supplementary Figures S3A, B). Among them, only 6 (6.6%) patients with TVR levels deteriorated ≥ 2 classes and the absolute level higher than moderate. These six patients had shorter average lead-TVA-dist (18.5 ± 9.6 vs. 20.9 ± 6.6 mm, *p* = 0.61) and significant longer follow-up time (23.3 ± 2.7 vs. 18.6 ± 6.6 months, *p* = 0.005) than the other patients, corresponding to the results above.

## 4 Discussion

There were several primary findings in our study: 1) in patients indicated for pacemaker implantation with LVEF > 40%, LBBP maintained stable and acceptable pacing and ECG parameters; 2) echocardiographic measurements revealed that the tip of the leads kept stable at the sub-endocardial area of LVS, and functional parameters remained stable or even slightly improved in the long-term follow-up period; 3) baseline LVEF, ∆ Paced QRSd and corrected longit-dist negatively and independently correlated with the change of LVEF, which might be the indicator for long-term potential LVEF decrease in these population.

In recent years, LBBP has been considered a novel and feasible pacing maneuver to achieve near-physiological pacing. The short-term feasibility and effectiveness of this technique have been demonstrated, with the advantages compared to HBP such as the shorter learning curve, the higher success rate, and rare perioperative complications (Chen et al., 2019; Li et al., 2020; Liu et al., 2021). Subsequently, several mid/long-term studies investigated the performance of LBBP, revealing that LBBP could maintain stable pacing and ECG parameters during the follow up (Padala et al., 2020; Sharma et al., 2021; Su et al., 2021). Our study provided further evidence to support these results. Through a detailed comparison of baseline and follow-up ECG, we observed a constant pacing performance with acceptable pacing parameters in most patients. Although the pacing impedance was within the clinically acceptable range and maintained around 400 Ω, the reduction in the pacing impedance was still prominent, which was also found in other studies (Chen et al., 2020; Su et al., 2021). For the traditional RVP, the decrease in impedance was usually accompanied by increased pacing threshold and/or decreased R wave amplitude and was considered to be suggestive of the abrasion of lead or the ventricular septal perforation. However, we thought the decreased impedance in LBBP might be related to the 3830 lead characteristics and the myocardial properties of the left bundle branch area, rather than as a sign of the lead wear or ventricular septal perforation. Still, the exact reason remains unclear and requires further confirmation. Only a few patients had signs suggesting degeneration of pacing performance, such as significant prolonged (>10 ms) paced QRSd (9.9%) and V6RWPT (7.8%), or disappearance of RBBB pattern in V1 ECG lead (4.4%). As patients’ data were collected during clinic visits, it was difficult to confirm LBB capture and judge the LBB capture threshold *via* normal speed (25 mm/s) ECG; Besides, there is no direct and established method or standard to determine LBB capture except electrophysiologic study, the transition of QRS morphology from s-LBBP or LVSP to ns-LBBP is not sensitive enough as it depends on the obvious discrepancy of capture threshold between myocardium and LBB.

In addition to the pacing and ECG parameters, the anatomic position stability of 3830 lead was also evaluated. In the study by Vijayaraman et al. (2019), echocardiography was performed in Left bundle branch area pacing (LBBAP) patients to assess the average intra-septal depth of the lead along the course of the lead, which was 1.4 ± 0.23 cm (range 1.1–1.8 cm). However, the distance from the tip to LVS was not assessed and whether the tip was located at the sub-endocardial area of LVS was not elucidated. In the study with the largest sample size of LBBP, the long-term stability of anatomic lead positions was not evaluated as well (Su et al., 2021). In the present study, echocardiographic measurement was performed at follow-up and revealed the median distance from the lead tip to LVS was 0.4 mm (0.8 ± 1.1 mm). This result demonstrated that most of the leads’ tip was stable at the sub-endocardial area of LVS.

LBBP could achieve favorable LV electrical and mechanical synchrony similar to HBP(8). Previous studies pointed out that LBBP could maintain or even improve cardiac function in the acute postoperative phase, and improve long-term clinical outcomes compared to traditional RVP, especially in patients with a high burden of ventricular pacing (Li et al., 2020; Li et al., 2021; Liu et al., 2021). In this series, the results demonstrated 29 (33%) patients had the ∆LVEF ≥ 5%, 48 (55.2%) had the ∆LVEF ranging from −5% to 5%, and only 10 (11.5%) had the ∆LVEF < −5%. These results indicated that the adverse effect of LBBP on long-term LVEF was small in patients without reduced cardiac function. The overall improvement of LVEF we reported was lower than prior studies. The population in our study had a better baseline cardiac function than patients enrolled in previous study, which limited the potential rise in LVEF.

Although a stable trend has been observed for long-term LVEF after LBBP, which group of patients is more likely to show an improvement or decline in LVEF remained unknown. As a result, comparison between patients with ∆LVEF > 0 and ∆LVEF ≤ 0 was performed to explored the potential correlated factors for LVEF changes in this population. The Logistic-Lasso regression model was applied to rule out the possible confounding effects of these factors, as it was considered as a more solid and scientific method of variable screen. Finally, the three-variable model was obtained with favorable fitness (AUC = 0.79), including baseline LVEF, ∆Paced QRSd, and corrected longit-dist, which were associated with LVEF decrease. Further, we demonstrated the negative linear correlation of these variables to ∆LVEF.

We believe that these results are clinically reasonable, as the greater baseline LVEF was, the smaller space remained for the long-term rise of LVEF, even a decrease of LVEF due to the progress of comorbidity or cardiac risk factors. Lower ∆Paced QRSd meant the paced QRSd was less prolonged, which correlated to a more stable and synchronized ventricular depolarization during follow-up, which might contribute to the maintenance or the improvement of cardiac function. Corrected longit-dist was proposed as a novel distance parameter for describing the position of LBBP lead (Lu et al., 2021). By eliminating the influence of inter-individual variations of cardiac dimension, corrected longit-dist could more accurately reflect the LBBP lead implanted at the upper or lower region of the IVS. Lower corrected longit-dist meant that the LBBP lead was implanted at the upper region of the IVS. Considering the LBB trunk is commonly located in the upper portion of the IVS, the lead with lower corrected longit-dist (upper IVS) was more likely to capture the LBB trunk; besides, pacing from the upper part of IVS might generate an electrical axis more similar to intrinsic rhythm. Therefore, we considered that the lead with a lower corrected longit-dist could make the paced ventricular depolarization more physiological by capturing the LBB trunk and generating a near-normal electrical axis, which was theoretically beneficial to the long-term cardiac function.

Moderate or greater TVR is associated with adverse RV function, increased risk of new-onset heart failure, and poor long-term survival (Topilsky et al., 2014; Papageorgiou et al., 2020). About 5%–21.7% of patients developed TVR deterioration after RV lead implantation (Van De Heyning et al., 2019; Papageorgiou et al., 2020), but the data after LBBP was rare. The patients with TVR deterioration had longer follow-up time and shorter Lead-TA-dist. However, the effects of LBBP on the tricuspid valve remained to be further validated.

## 5 Study limitations

As a single-center and relatively small-sample observational study in patients undergone LBBP for symptomatic bradycardia, there were inevitable observational bias and lower statistical power. The follow-up intervals of echocardiography were not consistent for all patients, which might also influence the results. However, the results supported that the pacing parameters, cardiac function, and lead’s anatomic position were stable after a relatively long-term period of LBBP. The absolute value of average LVEF improvement was small since patients included in the study had a relatively normal cardiac function. Although we found the potential factors correlated to ∆LVEF which were insufficient to construct a clinical prediction model for cardiac function improvement or deterioration, these results still possessed referential value in clinical practice. Studies with larger sample size and prolonged follow-up period are required to confirmed the results.

## 6 Conclusion

The results from this single-center prospective observational study supported the long-term stability of LBBP regarding pacing performance, the anatomic position of the leads, and patients’ cardiac function, indicating the long-term safety and feasibility of LBBP in bradyarrhythmia patients. Besides, the baseline LVEF, ∆ Paced QRSd and corrected longit-dist were potential factors correlated to long-term changes of LVEF, while patients with TVR deterioration had longer follow-up time and shorter Lead-TA-dist, which required further confirmation.

## Data Availability

The raw data supporting the conclusion of this article will be made available by the authors, without undue reservation.
