# Supplementary material for: Long-term follow-up results of patients with left bundle branch pacing and exploration for potential factors affecting cardiac function
Source: Front Physiol. 2022 Sep 15;13:996640. doi: 10.3389/fphys.2022.996640 (PMC9520459; doi:10.3389/fphys.2022.996640)

frontiers

Supplementary Material

Supplementary Table 1. Definitions of distance parameters.

Distance parameters	definition
Contraction line (CL)	the tricuspid annulus and the contraction point of pulmonary artery segment (the intersection point of paradoxical movements during pulmonary artery emptying and ventricular diastole, also the zero point of the coordinate system) were identified in the dynamic fluoroscopic imaging. The lower end point of CL was the junction point of the tricuspid annulus plane and the lower cardiac outline. The upper end point of CL was the zero point.
Distance from CL to apex (CL-apex-dist)	the vertical distance from CL to cardiac apex in ventricular end-diastolic phase.
Longitudinal distance (longit-dist)	the vertical distance from the tip of the 3830 lead to CL in ventricular end-diastolic phase; The longit-dist of sites on the near-apex side of CL was positive while the value was negative on the near-cardiac-base side.
Lateral distance (lat-dist)	the distance from the tip of the 3830 lead to the line perpendicularly passing through the upper endpoint (the zero point) of CL.
Corrected longit-dist	In order to eliminate the influence of inter-individual variations of cardiac dimension, corrected parameters were created. The corrected longit-dist of a particular lead-implanted site was calculated by dividing its longit-dist by its CL-apex-dist then multiplied by the mean CL-apex-dist of all enrolled sites.
Corrected lat-dist	The corrected lat-dist of a particular lead-implanted site was calculated by dividing its lat-dist by its length of CL then multiplied by the mean length of CL of all enrolled sites.

A novel coordinate system was invented on the ventricular end-diastolic fluoroscopic image in RAO 30° view to describe the distribution of the lead-implanted sites quantitatively. Conversion from the measured distance to the actual distance was achieved via the bridge of the 3830 lead's diameter. In each fluoroscopic image, the actual distance was calculated by dividing the measured distance by the measured diameter of 3830 lead then multiplied by 1.4 mm — the actual diameter of 3830 lead. The diameter of the 3830 lead was measured at three different segments of the lead and the average was taken.

Supplementary Table 2. Comparison between patients with improved and decreased LVEF.

Variables	ΔLVEF > 0 (n=59)	ΔLVEF <= 0 (n=28)	P value
Age (years)	67 (61, 73)	62 (52.5, 72)	0.13
Male sex	23 (39.0)	15 (53.6)	0.20
Pacing indications (SSS/AVB/AF bradycardia)	24/33/2 (40.7/55.9/3.4)	11/15/2 (39.3/53.6/7.1)	0.73
Intrinsic QRS duration (ms)	90 (84.8, 99.4)	87.5 (84.3, 95.3)	0.50
Intrinsic QRS morphology (narrow/LBBB/RBBB)	49/4/6 (83.1/6.8/10.2)	24/0/4 (85.7/0/14.3)	0.39
Baseline (BL)			
Paced QRS duration (ms)	103.1 ± 13.0	106.9±9.1	0.11
V6RWPT (ms)	69.1±10.0	66.4±9.1	0.21
V1RWPT (ms)	101.2±12.1	100.4 ±9.1	0.73
V6-V1 interpeak interval (ms)	32.0±10.5	34.0±8.5	0.35
LVEF (%)	62 (60, 65)	65 (62, 65.3)	0.003
Follow-up (FU)			
Paced QRS duration (ms)	102.4±13.2	111.7±8.6	0.0002
V6RWPT (ms)	71.2±9.3	70.8±10.9	0.88
V1RWPT (ms)	102.9±11.6	105.4±8.4	0.27
V6-V1 interpeak interval (ms)	31.8±11.2	34.7±9.6	0.24
LVEF (%)	67.6 (65, 70)	60 (58, 62.1)	<0.0001
Changes between BL and FU			
ΔPaced QRS duration (ms)	-0.4 (-5.1, 4.7)	2.5 (-0.7, 9.3)	0.006
ΔV6RWPT (ms)	2.0 (-0.6, 5.6)	5.1 (0.4, 6.9)	0.04
ΔV1RWPT (ms)	1.2±7.3	4.6±6.0	0.03
ΔV6-V1 interpeak interval (ms)	-0.3 ± 6.7	0.1±6.1	0.78
VP>40%	30 (50.8)	13 (46.4)	0.70
Follow-up time	17 (13, 22)	18.5 (14.5, 24)	0.40
Distance parameters			
Lead-TA-dist (mm)	20.1±7.2	22.4±5.7	0.11
Lead depth in IVS (mm)	10.7±2.0	11.2±2.3	0.34
Lead tip to LVS (mm)	0.3 (0, 1.4)	0.4 (0, 1.6)	0.93
Length of CL (mm)	146.6 (139.3)	148.9 (158.4)	0.95
CL-apex-dist (mm)	199.3±12.9	119.0±10.5	0.93
Corrected longit-dist (mm)	23.4±11.0	29.1±10.5	0.02
Corrected lat-dist (mm)	80.1±13.3	77.8±12.5	0.44

Data was presented as n (%), mean±SD, or median (IQR). LVEF, left ventricular ejection fraction; LBBB, left bundle branch block; RBBB, right bundle branch block; V6RWPT/V1RWPT, stimulus to R wave peak time in V6/V1 ECG lead; ΔPaced QRSd/V6RWPT/V1RWPT , changes of QRSd/V6RWPT/V1RWPT from baseline to follow-up; VP, ventricular pacing proportion; Lead-TA-dist, distance from the lead implanted site to the tricuspid annulus; IVS, interventricular septum; LVS, left surface of ventricular septum; CL, contraction line; CL-apex-dist, distance from CL to apex; Longit-dist, longitudinal distance; Lat-dist, lateral distance.

Supplementary Table 3. Comparison between patients with and without TVR deterioration.

Variables	TVR deterioration (n=32)	TVR not deterioration (n=59)	P value
Age (years)	67 (58.8, 73.5)	66 (58.5, 73.0)	0.50
Male sex	11/32 (34.4)	28/59 (47.5)	0.23
Intrinsic QRS duration (ms)	87.5 (84.4, 95.4)	89.8 (84.3, 96.9)	0.78
Intrinsic QRS morphology (narrow/LBBB/RBBB)	26/1/5 (81.2/3.1/15.6)	51/3/5 (86.4/5.1/8.5)	0.57
Baseline (BL)			
Paced QRS duration (ms)	106.2±14.0	103.9±10.6	0.43
V6RWPT (ms)	67.5±8.9	68.5±10.2	0.63
V1RWPT (ms)	100.0±10.5	101.4±11.5	0.54
V6-V1 interpeak interval (ms)	32.5±11.0	32.9±9.5	0.84
LVEF (%)	62 (60, 65)	63 (60, 65)	0.49
Follow-up (FU)			
Paced QRS duration (ms)	106.0 ± 13.5	105.5 ± 12.1	0.85
V6RWPT (ms)	69.4 ± 9.1	72.0 ± 10.2	0.22
V1RWPT (ms)	101.2 ± 9.8	105.0 ± 11.0	0.11
V6-V1 interpeak interval (ms)	32.0 ± 10.8	32.9 ± 10.8	0.72
LVEF (%)	65.5 (63, 67)	65 (61, 69)	0.64
Changes between BL and FU			
ΔPaced QRS duration (ms)	0.3 (-3.8, 6.1)	0.2 (-3.1, 6.1)	0.89
ΔV6RWPT (ms)	1.9 (-0.7, 5.1)	4.15 (0.2, 6.7)	0.09
ΔV1RWPT (ms)	1.7 ± 0.6	2.5 ± 7.2	0.60
ΔV6-V1 interpeak interval (ms)	-0.3±6.1	-0.4 ± 6.6	0.89
ΔLVEF	2.3 ± 4.8	2.7 ± 6.8	0.74
ΔTVR flow speed (m/s)	1.8 (0.1, 2.4)	-0.2 (-0.4, 0)	<0.01
ΔTVR pressure gradient (mmHg)	12.2 ± 15.5	-5.9 ± 9.4	<0.01
VP>40%	14/32 (43.8)	30/59 (50.8)	0.52
Follow-up time	20.5 (17.0, 24.0)	15.0 (12.5, 21.5)	0.01
Distance parameters			
Lead-TVA-dist (mm)	18.6 (14.0, 23.0)	21.6 (18.9, 25.8)	0.04
Lead depth in IVS (mm)	10.7±1.4	10.9 ± 2.3	0.51
Lead tip to LVS (mm)	0.4 (0, 1.3)	0.3 (0, 1.6)	0.60
Length of CL (mm)	147.6 (142.5, 155.7)	147.5 (139.3, 155.5)	0.71
CL-apex-dist (mm)	117.4 ± 13.9	119.1 ± 12.2	0.58
Corrected longit-dist (mm)	26.2 ± 10.4	25.2 ± 11.5	0.68
Corrected lat-dist (mm)	78.1 ± 13.5	80.1 ± 13.2	0.49

Data was presented as n (%), mean±SD, or median (IQR). LVEF, left ventricular ejection fraction; LBBB, left bundle branch block; RBBB, right bundle branch block; V6RWPT/V1RWPT, R wave peak time in V6/V1 ECG lead; ΔPaced QRSd/V6RWPT/V1RWPT , changes of QRSd/V6RWPT/V1RWPT from baseline to follow-up; VP, ventricular pacing proportion; Lead-TA-dist, distance from the lead implanted site to the tricuspid annulus; IVS, interventricular septum; LVS, left surface of ventricular septum; CL, contraction line; CL-apex-dist, distance from CL to apex; Longit-dist, longitudinal distance; Lat-dist, lateral distance.

Supplementary Figure 1. Flow-chart of patient selection.

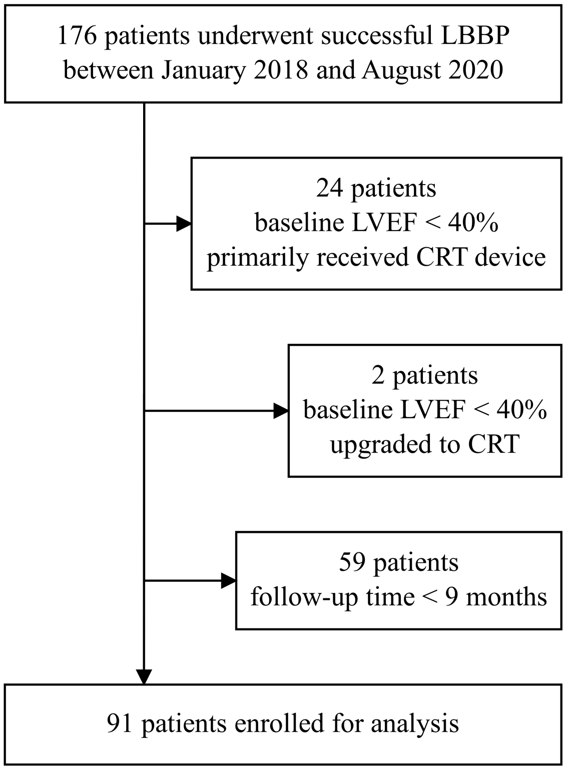

Supplementary Figure 2. Normal distribution diagnosis for the three linear regression models.

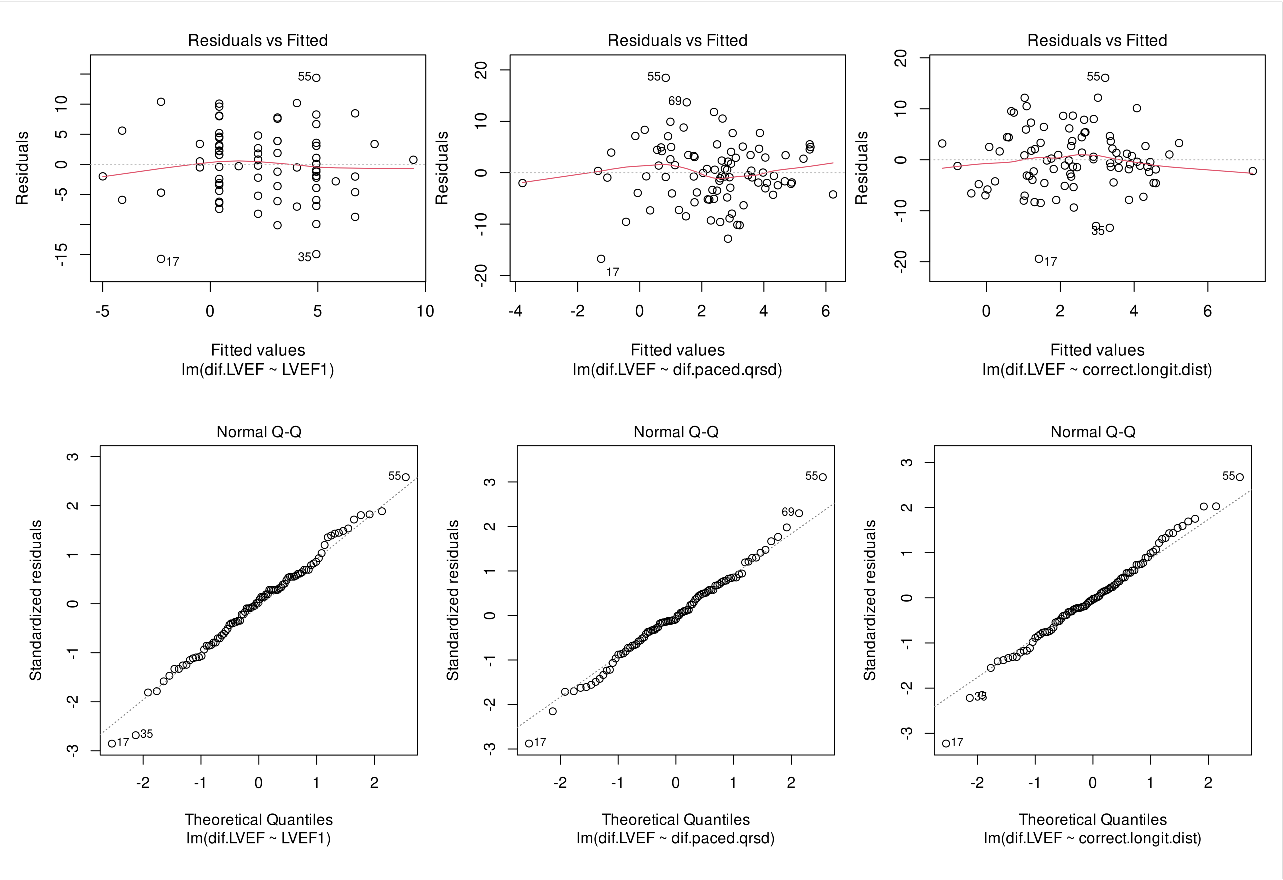

Supplementary Figure 3. Comparison between patients with and without TVR deterioration. (A) The follow-up time was significant longer in patients with TVR deterioration. (B) Lead-TA-dist was significant shorter in patients with TVR deterioration. TVR, tricuspid valvular regurgitation; Lead-TA-dist, distance from the lead implanted site to the tricuspid annulus.

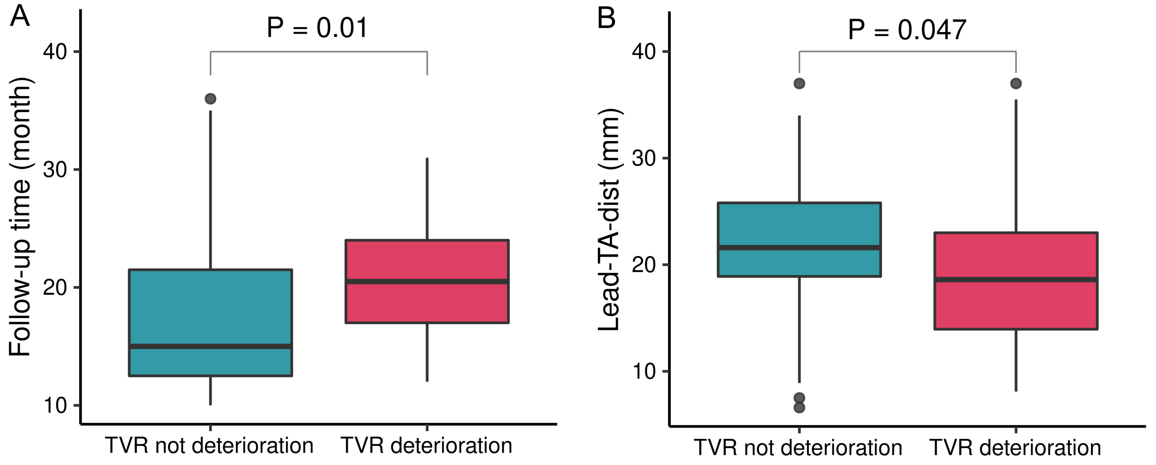

Supplement: Supplementary file 1 [file DataSheet1.PDF]
